# Turning the trolley with reflective equilibrium

**DOI:** 10.1007/s11229-022-03762-3

**Published:** 2022-06-24

**Authors:** Tanja Rechnitzer

**Affiliations:** Institute of Philosophy, Leibniz University Hannover, Lange Laube 6, Hannover, 30159 Germany

**Keywords:** Reflective equilibrium, Trolley problem, Philosophical methods, Methodology of ethics, Reconstruction

## Abstract

Reflective equilibrium (RE)—the idea that we have to justify our judgments and principles through a process of mutual adjustment—is taken to be a central method in philosophy. Nonetheless, conceptions of RE often stay sketchy, and there is a striking lack of explicit and traceable applications of it. This paper presents an explicit case study for the application of an elaborate RE conception. RE is used to reconstruct the arguments from Thomson’s paper “Turning the Trolley” for why a bystander must not divert a runaway trolley from five workmen onto one. Analyzing Thomson’s resulting position with the RE-criteria has two main results: Firstly, the adjustment of one of her commitments can be defended. Secondly, no justified position in RE was reached. With respect to RE as a method, the main results from this application are: (1) There is at least one conception of RE that is sufficiently specified to be applicable; (2) the RE criteria put real constraints on the process of justification; and (3) an explicit application of RE has benefits in terms of clarity while at the same time providing guidance for how the justificatory process could be continued.

## Introduction

Should you kill one person in order to save five? This question is at the center of the debate around so-called “trolley cases”. To find a justified answer, one might try to use the method of reflective equilibrium (RE), which is taken to be a central method of philosophy (e.g., Lewis, 1983, Introduction; Scanlon, 2003, p. 149). Its basic idea is that we have to start from our existing judgments about relevant cases and search for principles that can account for them. Both principles and judgments are adjusted with respect to each other in a process of reflective balancing, until a coherent whole—an equilibrium—is reached.

However, RE also has faced harsh criticism, such as being too weak to put real constraints on the justification process (Kelly & McGrath, 2010), being methodologically irrelevant (McPherson, 2015), or being trivial and uninformative [(Williamson, 2007), p. 244]. As Foley (1993, p. 128) sees it, the idea of RE is unhelpful:

It tells you essentially this: take into account all the data that you think to be relevant and then reflect on the data, solving conflicts in the way that you judge best. On the other hand, it does not tell you what kinds of data are relevant, nor does it tell you what is the best way to resolve conflicts among the data. It leaves you to muck about on these questions as best as you can.

Yet, critics often direct their attacks towards vague or underdeveloped conceptions of RE that have never been tested through actual application. Indeed, in general there is a striking lack of explicit applications of RE. This does not entail that no one really claims to apply reflective equilibrium, nor that no one has ever really applied it. However, typically either (1) the adopted conception of RE is sketchy, saying little more than that we have to mutually adjust our moral commitments and principles until a coherent position is reached, or (2) the available implementations of RE do not allow us to clearly identify and classify its elements, to track the adjustments that were made, and to assess the contributions of the RE criteria to the process of adjustments.^1^ Arguably, as long as these issues are not addressed, discussions about the merits of applying RE are idle. To fill this gap, this paper presents an explicitly traceable application of a rigorous account of RE, demonstrating that RE can work as a method of (moral) justification.^2^

My aim is to show, firstly, that it is possible to use RE to structure moral argumentation, secondly, that the RE criteria put real constraints on the justification process, and thirdly, that applying RE in such a way provides us with a better understanding of the problem at hand. In order to do so, I adopt an elaborate RE-conception and use it to reconstruct an argument arising from the debate about so-called trolley cases. The paradigmatic trolley case is one in which, if you do not act, five people will die, and if you do act, one person dies but the other five are saved. Depending on how the details are spelled out—e.g., do you have to divert a threat towards the single person, or do you have to push him to his death?—it seems permissible to kill one to avoid the death of five in some situations, but in other situations, it seems clearly unacceptable. But why, and on what grounds, can we justify these diverging judgments? By an exemplary reconstruction of the arguments in Thomson’s (2008) paper “Turning the Trolley” I demonstrate the fruitfulness of RE as a methodological framework to answer such questions.

The structure of this paper is as follows: Sect. 2 introduces the RE-conception that I adopt for the purpose of this paper. Section 3 describes the main results of my reconstruction of Thomson’s arguments as an RE-process. By analyzing her resulting position with the RE-criteria, I can show that while her adjustment of one of her commitments can be defended, no justified position in RE was reached. In Sect. 4, I discuss what these results mean for the applicability of RE and its uses as a method. Lastly, I summarize my main results in Sect. 5.

While the paper gives an overview of the general structure of the reconstructed RE-process and its main aspects, describing every detail is beyond its scope. If readers are interested in this, they should consult the appendix starting on p. 39. It includes a list of all the elements of the RE process and, together with the schematic overview given in Fig. 3, can be used to trace the whole process in detail. In any case, having the list of elements ready to consult while reading will also be useful to readers who are only interested in the main points.

## Reflective equilibrium

Roughly, the core idea of reflective equilibrium (RE) is that we have to start from our existing judgments about relevant cases and search for fitting systematic principles, which, in turn, can be applied to new cases. When conflict arises between judgments and principles, both have to be adjusted mutually in a process guided by pragmatic-epistemic aims and supported by accepted background theories. When a coherent whole—an equilibrium—is reached as a result of this process, then we can consider both our judgments and our principles to be justified. The term *reflective equilibrium* was coined by Rawls (1999), but the idea goes back to Goodman’s (1983) investigation of the justification of logics. In this paper, I adopt the RE-conception of Brun (2013, 2020) and Baumberger and Brun (2017, 2021), who developed the most elaborate and rigorous RE-conception to date by building on the works of Goodman, Elgin (1996, 2017), and Carnap.

Standard accounts of RE typically see the difference between judgments and principles in terms of particular vs. general, but Rawls (1974, p. 289) had already pointed out that judgments can also be general. Hence, Brun (2013, p. 240) argues that the main difference between principles and judgments has nothing to do with their content or their form, but that it is their function that is different (see also Knight, 2017, 52). He argues that principles are *part of a system*, while judgments involve a certain degree of *commitment*—as minimal as it may be. Together, a set of commitments and a system form a *position*; this is the object of the justification via RE, i.e., both have to be justified together and contribute to each other’s justification. In order to provide a systematic account of our commitments, the system should possess theoretical virtues like accuracy, consistency, scope, simplicity, and fruitfulness (Kuhn, 1977, pp. 321–322).

We can distinguish between the *method* of reflective equilibrium, the *process* of trying to reach a reflective equilibrium, and the *state* of being in reflective equilibrium. Depending on the stage of the RE process, input, current, and resulting commitments can be distinguished.^3^

A position is in a state of reflective equilibrium—i.e., justified—when the following criteria are fulfilled:

1. The resulting commitments and the system are in agreement, i.e., they are consistent and the commitments are supported by the system;
2. the resulting commitments respect the input commitments adequately;
3. (at least some of) the resulting commitments have independent credibility;
4. the system does justice to the relevant theoretical virtues;
5. the resulting position—i.e., the resulting commitments and the system—is supported by background theories; and
6. the resulting position is at least as plausible as all available alternatives.

**Fig. 1:**
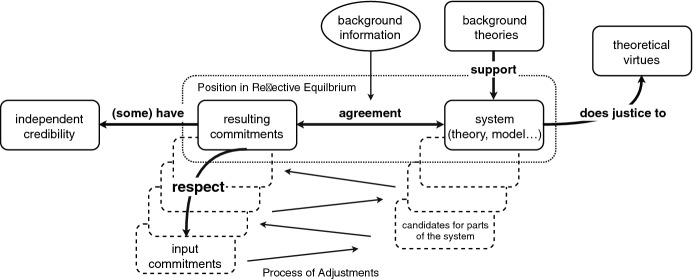
A Schematic Overview of Reflective Equilibrium

The relation of “support” mentioned in conditions (1) and (5) could be an inferential, explanatory, or probabilistic relation. Generally, something more than mere consistency is needed, but it does not always have to amount to strict deductive inference. Together, these criteria demand that the position is both internally and externally coherent. As for condition (2), the resulting commitments have to respect the input commitments in the sense that, looking back, a plausible argument can be given for every adjustment of an input commitment that was made on the way to the resulting commitments. This condition blocks the strategy of formulating, e.g., a very simple system with a broad scope and rejecting every commitment that conflicts with it. It thereby ensures that we do not change the subject (Brun, 2020, p. 937). Condition (3) shows that the justification of RE is not created *ex nihilo*: we have our input commitments because we ascribe some *independent credibility* to them, i.e., credibility they have independently of being justified via RE. Reasons for ascribing independent credibility to a commitment can be, e.g., that it accommodates some evidence—like observations, intuitions, or testimony—or that it can be supported by background theories (Baumberger and Brun, 2017, p. 177). To be part of a position in RE, resulting commitments should also have some degree of independent credibility in order to contribute to the credibility of the resulting position. Conditions (2) and (3) are closely connected, because one aspect of respecting input commitments is that in order to defensibly adjust a commitment, its independent credibility has to be outweighed by other considerations. Condition (6) is necessary because there will often be different ways to resolve conflicts, and thus there can be a plurality of RE-states that might be reached in different ways. Equally plausible positions are justified to the same degree, but should of course be given up in favor of more plausible positions (Elgin, 1996, p. 119), i.e., positions that meet the RE-criteria to a higher degree. Figure 1 gives a schematic overview of the main elements and requirements of RE.

If these conditions are met, then the resulting position is in a state of RE. It is worth pointing out, however, that there is no guarantee that such an RE-state can be reached. It is entirely possible that the RE process does not result in a justified position, i.e., a position that meets the RE-criteria. And even if such a state is reached, it will always be preliminary: New considerations or arguments might arise that make it necessary to continue the process of adjustments (Rawls, 1999, p. 18). But how should we proceed in order to work towards a position that is in reflective equilibrium? I propose to spell out the process in terms of a method that is guided by the criteria for reaching an RE-state.

***Structuring the Process*** For the purpose of applying RE as a method, I propose to characterize the equilibration-process as starting from an initial position and proceeding in two alternating kinds of steps of “going back and forth” (Rawls, 1999, p. 18) between the set of commitments and the system. Identifying steps in which the RE-process proceeds—what Beisbart et al. (2021, p. 449) call “adjustment rules” and a “stopping condition”—is intended to provide structure to the case study and to help to explicitly trace the contribution and constraints of the RE criteria.

- *Step A, Adjusting the System* Holding the commitments constant, the system is adjusted with respect to the goals of increasing (i) agreement with the current commitments, and (ii) the theoretical virtuousness of the system.
- *Step B, Adjusting the Commitments* Holding the system constant, current commitments are adjusted in order to (i) increase agreement with the current system while (ii) respecting input commitments and (iii) selecting a set of commitments with a high independent credibility.

In both kinds of steps, the different desiderata might not always be compatible. In instances of Step A, increasing agreement with current commitments will often decrease the theoretical virtuousness of the system, e.g., by making it more complex. Similarly, in instances of Step B, increasing agreement with the system—and thereby increasing the credibility of the position as a whole—often might speak against preserving commitments that on their own have a high independent credibility. In such situations, there are often no clear-cut criteria available to resolve the trade-offs. Ultimately, they often can only be defended retrospectively, i.e., based on whether the individual decisions taken did lead to an overall more plausible position.

The set of commitments might be adjusted because new commitments are inferred from the system, because current commitments are given up, or because formerly implicit commitments are made explicit. This makes it necessary to add one further distinction, namely, between *initial* input commitments and *emerging* input commitments (Baumberger & Brun, 2021). Initial commitments are the sub-class of the input commitments that the RE-process initially starts out with.

Emerging commitments are another subclass of the input commitments. They are commitments which are made explicit during the RE-process but not because they are inferred from the system, and neither because they are a direct result of an adjustment to the position. This might happen because during the RE process, we can be confronted with new considerations that never occurred to us before.

The RE-process comes to an end point when neither of the two steps brings any improvement to the position—this is the “stopping rule”. However, such an end point does not necessarily mean that the position is in reflective equilibrium, it just means that the position can no longer be improved towards reaching RE. We then have to ask whether, and to which degree, the RE-conditions (1)–(6) are fulfilled.

## Reconstructing ‘turning the trolley’ as an RE-process

The trolley problem was selected for the case study because it is one of the paradigmatic examples of a problem of moral justification. Thomson’s (2008) paper “Turning the Trolley” was chosen because it has a manageable number of cases, includes explicit proposals for principles, and results in a reversal of a formerly held commitment. In my reconstruction of Thomson’s arguments as an RE-process, I focus on the reversal of this commitment. Thus, I leave out smaller details that can also be reconstructed in RE but that do not directly contribute to the discussion of the bystander case. For example, the case of *Car Driver’s Three Options* (Thomson, 2008, pp. 370–371) will not be addressed.

The goal of the reconstruction is to provide a charitable reconstruction of Thomson’s arguments, and to assess whether her results can be defended based on the RE-criteria. As this is a reconstruction, it is not necessary to follow Thomson’s paper chronologically. Instead, I take her points into account when it makes the most sense from an RE-perspective, e.g., adding a principle to the system at a point where this can be defended based on its contribution to the degree of agreement with the current commitments. Sometimes I introduce further case variations, commitments, or principles that are in line with Thomson’s other remarks and add to the plausibility of her arguments, but are not explicitly made by her. These additions are clearly marked as such in the appendix (see p. 39), where all the material that enters the RE-process is listed and references are given.

In the remainder of this section, I first describe the initial position with the conflict that gets the RE-process going in 3.1. I then describe key points from my reconstruction of Thomson’s arguments as eight steps of an RE-process in 3.2, before analyzing her resulting position with the RE-criteria in 3.3.

### The initial position

#### Cases, commitments, and principles

The initial position consists of five commitments and two principles. Four of the commitments concern a rather narrow subject matter, focusing on cases in which we have to choose between the lives of five people against the life of one person.

In Case 1, *Judge’s Two Options*, a judge faces the decision between framing an innocent person for a crime she did not commit, and having her executed, versus letting rioters kill five hostages. In Case 2,*Trolley Driver’s Two Options*, the driver of a runaway trolley faces the decision between letting his trolley continue on the straight track, where it will run over five workmen, or diverting it to the right, where only one workman will die. In Case 3, *Bystander’s Two Options*, a bystander has the option of turning a driver-less runaway trolley from the straight track, where it would kill five workmen, to the right, where only one workman would die. In Case 4, *Fat Man*, the bystander finds himself standing on a footbridge over a track, where a runaway trolley threatens to kill five workmen, and realizes that he could push the large man that is standing next to him off the bridge, which would kill the fat man but save the five workmen (all case descriptions can be found in more detail and including references in the appendix).

Thomson’s initial commitments (IC) regarding these cases are:

- *IC 1* In Case 1, the judge must not frame an innocent person and have her executed to save five hostages.
- *IC 2* In Case 2, the driver of a runaway trolley must divert his trolley so that one workman dies instead of five.
- *IC 3* In Case 3, the bystander at the switch may divert the trolley so that one workman dies instead of five.
- *IC 4* In Case 4, the bystander on the bridge must not shove the fat man in front of the trolley in order to save the five workmen on the track.

Thomson assumes that most people would share these commitments, which, from an RE-perspective, would mean that they have a high independent credibility. I am not going to problematize this at this stage of the reconstruction, but come back to it when evaluating the resulting position in 3.3.

According to Thomson, the crucial difference between *Trolley Driver’s Two Options* and *Judge’s Two Options* is the following: the trolley driver would kill the five workmen on the straight track if he did not divert. But the judge would not kill the five hostages: he merely would not interfere, i.e., he would let them die. But in executing an innocent person, he would kill her. Thus, Thomson suggests two first principles (P) (Thomson, 2008, p. 360):

- $P\,1:$ *Letting Five Die Vs. Killing One Principle*] A must let five die if saving them requires killing B.
- $P\,2:$ *Killing Five Vs. Killing One Principle*] A must not kill five if he can instead kill one.

Those principles are intended as *ceteris paribus* principles, since further information about, e.g., the potential victims, might make a difference.

Thomson (2008, p. 360) sees these principles as grounded in the distinction between positive and negative duties, i.e., that what we owe other people in form of non-interference (negative duties) outweighs what we owe to them in form of active interference (positive duties). Thomson is committed to this distinction throughout the paper, and I thus added it as IC 5. This commitment becomes relevant again in Step $\hbox {A}_4$ (see p. 27), when it is explicitly introduced as a principle.

#### The initial conflict

**Fig. 2:**
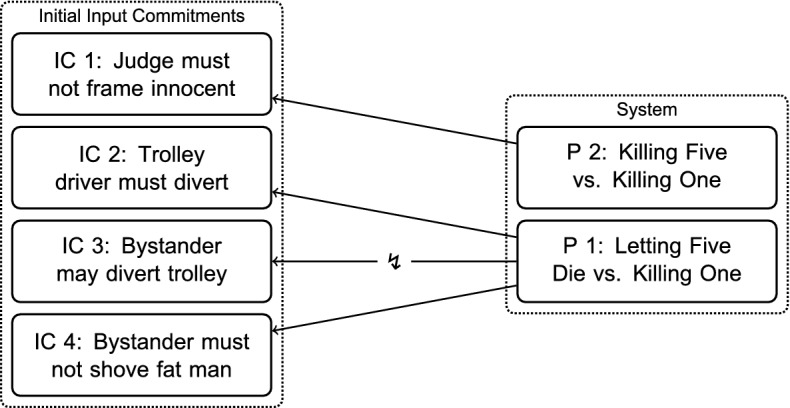
The Initial Position: Conflict between a Principle and a Commitment

The initial position is inconsistent, as shown in Fig. 2. The two principles are in agreement with IC 1, 2, and 4, represented through a solid arrow. However, represented through the interrupted arrow, there is a conflict with IC 3: the bystander would let the five on the straight track die if he did not divert the trolley. But by actively diverting the trolley, he would kill the one workman on the other track. Consequently, P 1, the *Letting Five Die Vs. Killing One* Principle, tells us that he must not divert the trolley.

From the perspective of RE, we now seem to have two obvious strategies to solve the conflict and establish consistency:

1. Rejecting the conflicting principle.
2. Giving up the conflicting commitment.

However, Thomson at this point does neither, but starts by broadening her set of commitments. Let me explain why, and how I reconstruct this as part of the RE-process.

***Strategy 1*** Rejecting P 1, the *Letting Five Die Vs. Killing One* Principle, would resolve the inconsistency. But it would also leave three out of our four commitments unsupported. We would then need to find one or more other principle(s) that can support them.

Nothing speaks in general against this strategy. In fact, in her earlier paper “The Trolley Problem” (Thomson, 1985), Thomson follows this strategy and rejects the conflicting principle. However, when writing “Turning the Trolley”, Thomson (2008, p. 363) rejects earlier solutions of the trolley problem as not satisfactory, which seems to include her own. As my goal is to demonstrate the applicability of RE via reconstructing “Turning the Trolley”, I follow Thomson’s verdict and do not further explore Strategy 1. Still, from an RE-perspective, it is important to stress that adjusting the system would be a legitimate way to proceed. In fact, that Thomson does not explore this route weakens her position, a point which I address in Sect. 3.3.

***Strategy 2*** The second strategy—reversing the commitment, accepting that the bystander must not turn the trolley, and instantly establishing consistency—looks like the least laborious solution. However, it cannot be vindicated by the RE criteria at this stage. The problem is that this conflicts with the criterion to respect input commitments. For Thomson, the bystander commitment, IC 3, has a high independent credibility: she has a strong intuition that the bystander *may* divert the trolley, and, according to Thomson (2008, p. 360) , most people agree on this.^4^ The current position does not outweigh this independent credibility: we are also committed to the judgment that the trolley driver must divert the trolley and kill one instead of five (IC 1). Can we really argue that it makes such a big difference whether the trolley is diverted by someone driving the trolley or by a bystander at a switch? Of course, in the *Fat Man*-case we are committed that the bystander must not push the man over the bridge and in front of the trolley. But which of the two cases is similar in relevant aspects to the *Bystander at the Switch*-case, *Fat Man* or *Trolley Driver*? Our current system with the two principles tells us that it is *Fat Man*, and that accordingly, the bystander at the switch must not divert the trolley. (Of course, the current system only tells us this together with relevant background information, in this case most prominently the distinction between killing and letting die.) But without further support for this systematization, it could also be an accidental generalization—maybe the bystander in the *Fat Man*-case must not push the man over the bridge because this actively infringes a very stringent right of his, whereas diverting the trolley is done by throwing a switch, which does not infringe anyone’s right.^5^ Thus, without further reasoning, we cannot defend reversing the commitment simply on the grounds that it would establish consistency.

***A Third Strategy***If these were the only two strategies available, the prospects to resolve the conflict in the initial position would look dim. However, having only five input commitments is a rather narrow base. One way to adjust the current set of commitments is also to broaden it by, e.g., systematically exploring morally relevant factors and differences (Brun, 2017). Arguably, this is the strategy that Thomson employs: she starts working towards solving the conflict by considering her commitments on variations of the original cases as well as new cases, and adjusting the system accordingly. In the next subsection, I describe central aspects of the reconstructed process of adjustments in more detail.

### The process of adjustments

#### Overview

**Fig. 3:**
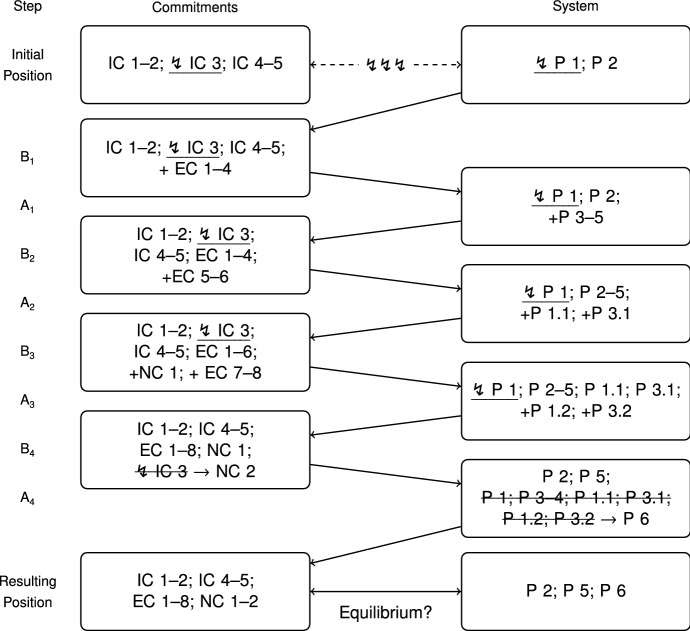
Schematic Overview of the Reconstructed RE-Process

For a schematic overview of my reconstruction of the process of adjustments, see Fig. 3. Together with the list of cases, commitments, and principles listed in the appendix, it can be used to trace the whole reconstruction. The initial position is inconsistent, as represented through the dashed line and the jagged arrows. As the schema illustrates, the process of adjustments proceeds in 8 steps (four B-steps and four A-steps) from the initial to the resulting position. As more and more emerging commitments (EC) are made explicit—often through considering systematic variations of the cases—more and more principles are introduced into the system in order to be able to support them. The direction of the process from one step to the next is indicated through the solid arrows. Jagged arrows next to an underlined commitment or principle are used to represent that this element is still in conflict.

Let me already point out two main results of the process. Firstly, in Step $\hbox {B}_4$, the conflict between IC 3 and P 1 is resolved: Thomson rejects the commitment that the bystander at the switch may turn the trolley onto the one workman, and accepts that he must not do so. That is, IC 3 is rejected and a newly inferred commitment is accepted instead, namely, NC 2. Secondly, in Step $\hbox {A}_4$ a large number of principles are reduced to P 6, the *Negative Duties Vs. Positive Duties Principle*. This significantly reduces the complexity of the system, while arguably it still is in agreement with all the current commitments: The resulting position is consistent. However, we still have to assess whether the resulting position is actually in a state of reflective equilibrium.

In the following, I do not describe the process and all its elements in detail, but zoom in on those aspects that are central in leading up to the reversal of IC 3.

#### Broadening the set of commitments

One strategy that Thomson uses repeatedly is to formulate variations of the cases. Within RE, we can interpret this as a form of Step B the set of current commitments is adjusted through exploring whether (a) Thomson has implicit input commitments on the case variations that can be made explicit, or, if not, whether (b) something can be inferred from the system for those cases. Commitments that “emerge” when considering other cases or exploring implications of commitments that we already hold count as input commitments in the same way as our initial commitments. Both groups are commitments that we hold independently of the RE-process, even if some of them only are made explicit during the process. Another way to put this is to say that emerging commitments are not resulting from the RE process, but could have, in principle, been discovered independently from it. Thus, both initial input commitments and emerging input commitments have to be respected by the resulting commitments in order for a position to be in reflective equilibrium. If we do not have any input commitments, but can infer something from the current system, we then have to decide whether or not we want to commit to these inferred judgments. Even if we commit to them, they do not count as input commitments; and the “respecting” condition does not apply to commitments that were newly inferred from the system and then became accepted just because of this.

For example, in Step $\hbox {B}_1$, Thomson’s most important variation is to put the bystander on his own piece of track in Case 5, *Bystander’s Three Options*, giving him the additional option of killing himself to save the five. If the bystander does not feel like dying today, but would still like to save the five, would it be permissible for him to turn the trolley onto the single workman (Thomson, 2008, p. 364)?

Thomson states that it would be unacceptable for him to impose onto someone else what he himself is not willing to do. Thus, the following commitment emerges:

- *Emerging Commitment (EC) 1* In Case 5, *Bystander’s Three Options*, it is unacceptable for the bystander to divert the trolley onto the single workman to save the five other workmen.

**Fig. 4:**
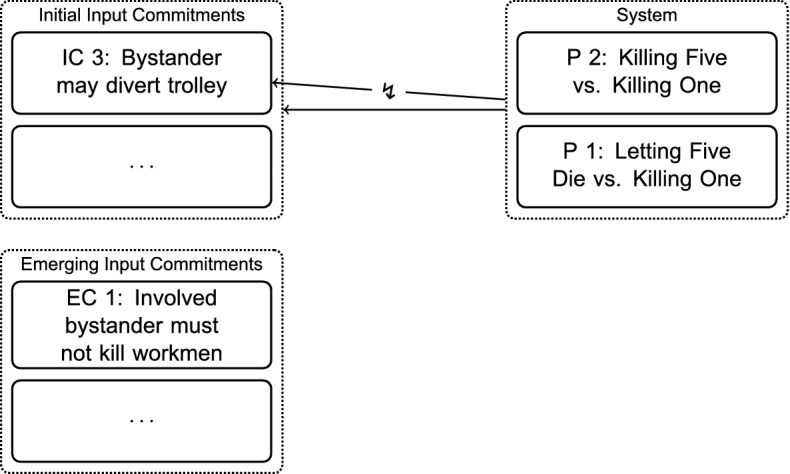
Adjusting Commitments: Emerging Input Commitments from Varying the Scenarios in Step $\hbox {B}_1$

Through considering the analogous Case 6, *Oxfam Donation* (see appendix), further commitments emerge at this step (EC 2–4). Figure 4 gives a schematic overview of the current position at the end of the first RE-step.

The solid arrow shows that the system is in agreement with most of the initial input commitments. However, there is still the conflict with the original bystander-case, which is signified by the interrupted arrow. The set of explicit input commitments has been broadened by considering variations of the scenarios. These are emerging input commitments, as they were not inferred in some way from the system. Currently, they are not directly supported by the system.

#### Adjusting the system through introducing new principles

As part of her argumentation, Thomson often formulates further principles, which are either specifications of existing principles or completely new ones. In the reconstruction, this is part of an instance of Step A. In those kinds of steps, the system is adjusted with respect to the two (not always compatible) goals of increasing (i) agreement with the current commitments, and (ii) the theoretical virtuousness of the system, e.g., through choosing simpler candidates over more complex ones. For example, in Step $\hbox {A}_1$, principles P 4 and P 5 are added in order to account for the emerging commitments from Step $\hbox {B}_1$, i.e., that the bystander with three options must not kill the one workman to save five (EC 1), but that neither is he required to kill himself to save them (EC 4).

- $P\,4:$ *Letting Five Die Vs. Killing One Vs. Killing Yourself Principle* A must not kill B to save five if A can instead kill A-self to save the five.
- $P\,5:$ *No Major Altruism Required Principle* One is not required to give up one’s life upon learning that five others will live if and only if one dies oneself.

**Fig. 5:**
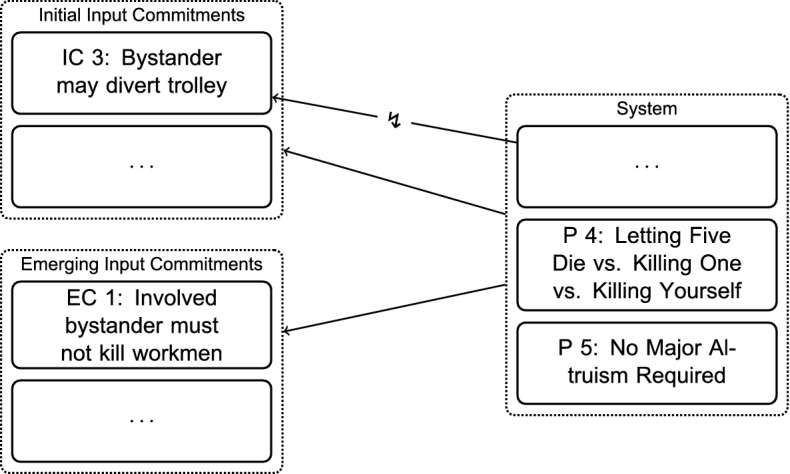
Adjusting the System: Formulating New Principles in Step $\hbox {A}_1$

As the schematic overview in Fig. 5 illustrates, the current system at the end of Step $\hbox {A}_1$ is in agreement with most of the current commitments. However, the conflict between P 1 and the initial bystander-commitment, IC 3, remains.

#### Fleshing out the position

The next four steps of the RE-process, $\hbox {B}_2$, $\hbox {A}_2$, $\hbox {B}_3$, $\hbox {A}_3$, continue to flesh out the position by making further commitments explicit through varying cases and drawing analogies, formulating new principles, and accepting implications based on the system as new commitments. In the following, I present the main points that are necessary to argue for the reversal of the initial bystander commitment, IC 3, in Step $\hbox {B}_4$.

After establishing that a bystander who could also kill himself to save the five must not kill the single workman to save them (Step $\hbox {B}_1$, EC 1), Thomson asks whether there is a relevant difference from the two options case: May a non-altruistic bystander, who would not be willing to sacrifice himself, kill the single workman—just because this bystander lacks the third option of killing himself (see Case 3.1 in the appendix)?

Thomson compares this with making someone else pay the cost of a good deed^6^, in this case, giving one’s life to save five: “Since [the non-altruistic bystander, T.R.] wouldn’t himself pay the cost of his good deed if he could pay it, there is no way in which he can decently regard himself as entitled to make someone else pay it” (Thomson, 2008, p. 366). As part of Step $\hbox {B}_2$, this reveals the following commitment:

- $EC\,6$ A non-altruistic bystander must not divert the trolley onto the single workman in Case 3.1, *Non-Altruistic Bystander’s Two Options*.

This still leaves open whether the bystander might be allowed to turn the trolley if he were an altruist. Consider Case 3.2, *Altruistic Bystander’s Two Options*. The altruistic bystander would be willing to sacrifice himself to save the five, if only he had the option. Would it be permissible for him to turn the trolley? Thomson does not have an immediate answer to this question, which illustrates that we do not always have precise and determinate commitments, but sometimes rather vague commitments of the sort “this case is relevant/our system should provide an answer here”.

In Step $\hbox {B}_3$, Thomson addresses the case of the altruistic bystander by first considering the single workman on the track. Is he required to be altruistic, i.e., to consent to be sacrificed to save the five? Again, we have no explicit input commitment on this case.

**Fig. 6:**
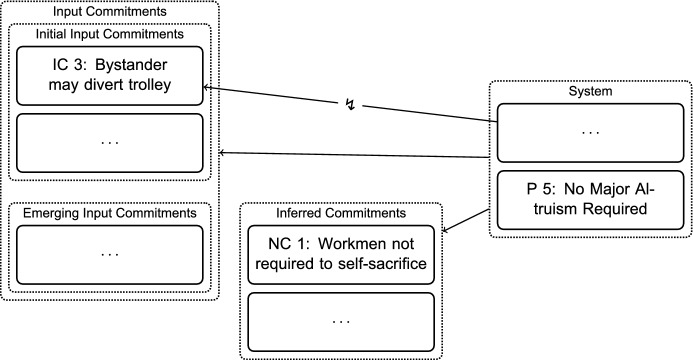
Adjusting Commitments: Adopting New Commitments Inferred with the System

The reconstruction of how Thomson answers this question illustrates another interesting aspect of RE: typically, a system will not only support the commitments we hold, but also—given background information—allow us to infer further propositions (see Fig. 6). In this situation, P 5, the *No Major Altruism Required Principle*, is relevant: according to P 5, it is permissible to let five people die if the only other means you have of saving them involves dying yourself. It follows that the single workman on the track is not required to give up his life to save the five. If we want to accept the system, this is something that we have to commit to. Given the other commitments that we hold, this seems reasonable. We thus have a newly inferred commitment (NC):

- *NC 1* The single workman on the track in *Bystander’s Two Options*^7^ is not required to consent to having the trolley turned onto him so that the five can be saved.

This means that the altruistic bystander cannot expect the workman to be equally altruistic, in two senses: Firstly, it is empirically unlikely that the workman will be such a major altruist who agrees to die to save five—we can support this with empirical background information. Secondly, the altruistic bystander cannot demand of the workman to consent to die, since the workman is not required to do so.

Thomson argues that under these circumstances, the altruistic bystander should choose the permissible option of letting the trolley continue^8^ over killing the workman (Thomson, 2008, p. 367):

- *EC 8* An altruistic bystander must not divert the trolley onto the single workman in *Bystander’s Two Options*.

In Step $\hbox {A}_3$ the system is adjusted by adding P 1.2, *Letting Five Die Vs. Killing One Principle, Altruist’s Version*, to account for the new commitment EC 8. The *Oxfam* analogy and the more general perspective on good deeds can be used to make this more plausible, leading to the introduction of another principle:

- $P\,3.2:$ *Second Modified Paying-For-Your-Own-Good-Deeds Principle* A must not make B pay the cost of a good deed that (i) A wants to do, (ii) A cannot pay the cost A-self, (iii) A would be willing to pay the cost A-selfs if possible, if B is not required to pay the cost.

Thus, as long as we accept Thomson’s presupposition that saving the five is a good deed, which would have the cost of one life, we can make the following argument^9^:

- The workman on the track is not required to sacrifice himself to save the five. (NC 2)
- The altruistic bystander wants to save the five. (Case 3.2)
- The altruistic bystander cannot pay the cost of saving the five himself. (Case 3.2)
- The altruistic bystander would pay the cost (killing himself) to save the five if possible. (Case 3.2)

Therefore, due to P 3.2, *Second Modified Paying For Your Own Good Deeds Principle*:

- The altruistic bystander must not divert the trolley onto the workman (EC 8).

We now are committed to the claim that neither a non-altruistic nor an altruistic bystander may turn the trolley in the *Two Options* case. We will use Step $\hbox {B}_4$ to consider the whole position in order to assess what this means for the conflict with the original bystander-commitment IC 3.

#### Reversing the commitment and solving the conflict

**Fig. 7:**
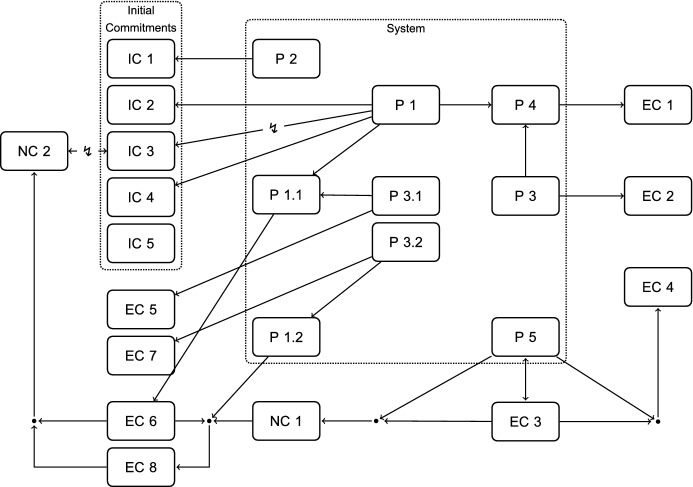
The Whole Current Position at the Beginning of Step $\hbox {B}_4$

The position has been fleshed out more and more. Compare Fig. 7 with Fig. 2, which respectively show the current position at Step $\hbox {B}_4$ and the initial position. The dots in Fig. 7 represent connecting arguments that are necessary to infer a commitment from the system. For example, EC 4, the commitment that the bystander with three options may let the trolley continue, is supported by P 5, the *No Major Altruism Required Principle*, through a connecting argument which is based on the description of Case 5, *Bystander’s Three Options*, and also includes EC 3, the commitment that one may refrain from doing a good deed if the only possible means of doing the good deed would require one to kill oneself. Solid arrows represent support, lines interrupted by a jagged arrow represent conflict.

While the conflict between P 1 and IC 3 persists between the initial position and the current position, the position has otherwise grown significantly. Inter alia, at the beginning of Step $\hbox {B}_4$, we are committed to the following:

- A bystander with the option of killing himself to save five must not kill someone else to save them. (NC 1)
- But: He is permitted to let five die instead of killing himself to save them. (EC 4)

And if the bystander can only choose between killing one workman or letting five die:

- A non-altruistic bystander must not kill one workman to save five. (EC 6)
- But: Neither must an altruistic bystander, who would be willing to kill himself to save five, kill someone else to save them. (EC8)

All of these commitments are in agreement with the current system, only the initial commitment on the original bystander case remains in conflict:

- *IC 3* The bystander at the switch may divert the trolley so that one workman dies instead of five. **[Conflicts with P 1]**

But if neither a non-altruistic nor an altruistic bystander may divert the trolley, then it seems plausible that no bystander may divert it. In the initial position, reversing IC 3 to establish consistency for the position could not be vindicated by the RE criteria: the condition of respecting input commitments was not fulfilled, since reversing an input commitment simply to establish consistency, and without further reasoning, is not defensible in RE. The independent credibility of the initial bystander commitment IC 3 was not outweighed in the initial position. But at the current point in the RE-process, we can provide further reasoning:

Firstly, the system has—by supporting further commitments—indirectly gained more weight. Stipulating that our intuitions on the different cases are about equally strong, each of the commitments it supports has a similar degree of independent credibility as the conflicting IC 3, and most of them are input commitments themselves.

Furthermore, the system supports commitments that are very similar to the conflicting commitment. Why should a not-further-characterized bystander be allowed to turn the trolley, as IC 3 states, while neither a non-altruistic bystander nor an altruistic bystander may do so? In the beginning, it was unclear whether the *Fat Man* or the *Trolley Driver’s Two Options* case is the relevant comparison to *Bystander’s Two Options*. But with Case 3.1 and 3.2, *Non-Altruistic Bystander’s Two Options* and *Altruistic Bystander’s Two Options*, it is hard to argue why there should be a relevant difference between them and the original “nondescript” bystander case.

All of this makes for a plausible reason to reject IC 3 and to accept a new commitment:

- *NC 2* The bystander in *Bystander’s Two Options* must not divert the trolley onto the single workman. [Replaces IC 3] [Inferred with P 1]

With this, the conflict in our position has been resolved. Did we reach a position that is in RE?

One might object that while the current system is in agreement with commitments that are similar to IC 3, this is through other principles than P 1, the principle that IC 3 conflicts with. Why does that speak in favor of accepting P 1’s verdict over IC 3? If you look again at Fig. 7, P 4, the *Letting Five Die Vs. Killing One Vs. Killing Yourself Principle*, as well as P 1.1, the *Letting Five Die Vs. Killing One Principle, Non-Altruist’s Version*, are specifications of P 1, i.e., specifications in the sense that they are restricting the *Letting Five Die Vs. Killing One Principle* to either altruistic or non-altruistic agents. Thus, via those specifications, P 1 does indeed support, e.g., EC 6 and EC 8, the commitments belonging to the (non-)altruistic bystander-cases.

Following the reasoning of RE leads me to add another Step, $\hbox {A}_4$, in order to make a suggestion on how the current system—consisting of 9 principles—can be simplified. This is not part of Thomson’s paper. But as she is committed to the distinction between positive and negative duties (IC 5), sees her principles as grounded in this distinction, and also comes back to it in the end of her paper when defending her results (Thomson, 2008, p. 372), it is a defensible addition to the reconstruction.

I have pointed out before that P 4 and P 1.1 are specifications of P 1, but the principle that negative duties outweigh positive duties whenever they conflict is even more general. Let us first introduce it explicitly as a further principle:

- *P* 6 :  *Negative Duties Vs. Positive Duties Principle* When a negative and a positive duty conflict, fulfilling the negative duty outweighs the positive duty.

That this principle is mostly a restatement of Thomson’s commitment to the difference between positive and negative duties (IC 5) demonstrates that the difference between commitments and system is one of function, not content or form (cf. Knight, 2017, 51-52). P 6 can support a large part of the resulting commitments, as long as we accept a range of distinctions, namely: that not killing someone is a negative duty, and saving someone from dying a positive one; that not stealing from someone is a negative duty, and making a donation to a good cause a positive one; that not making someone else pay for your good deeds without their consent is a negative duty, and doing a good deed a positive one.

**Fig. 8:**
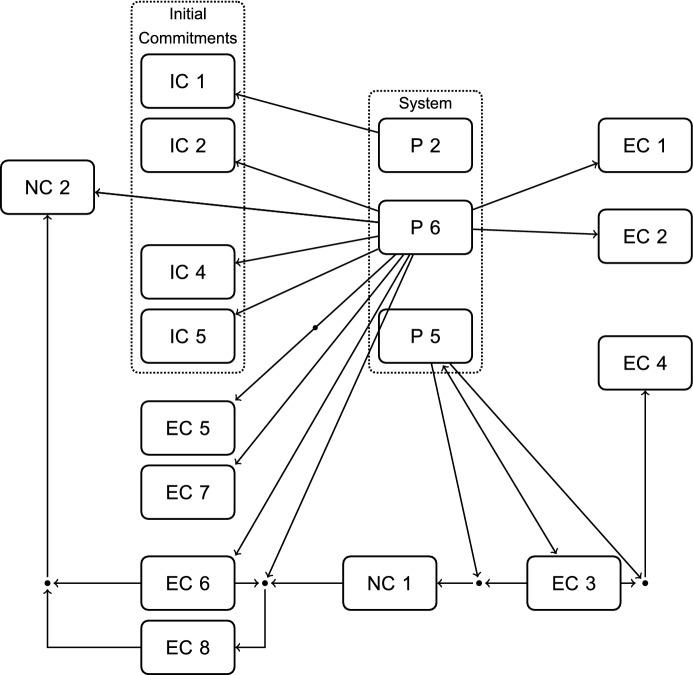
Step $\hbox {A}_4$: Simplifying the System

This, in turn, highlights the relevance of those distinctions. That there is a clear distinction, e.g., between killing and letting die, that can be made in cases like *Trolley Driver’s Two Options* and *Bystander’s Two Options* is something that Thomson presupposes. It is part of the background and plays a role in structuring the case descriptions. By making this explicit, the reconstruction also shows that parts of the background do important work for the argument.

*If* we accept all these distinctions in the background, though, we arrive at a much more systematic picture, with three instead of ten principles, as Fig. 8 illustrates. These principles are P 2, the *Killing Five Vs. Killing One Principle*, P 5, the *No Major Altruism Required Principle*, and P 6, the *Negative Duties Vs. Positive Duties Principle*. Together—through various specifications of P 6—these three principles support all of the resulting commitments.

In the next section, I discuss (a) whether it is plausible that the RE-process should come to an end here, i.e., whether none of the two steps could further improve the position, and (b) whether the resulting position is in a state of reflective equilibrium, i.e., whether the RE-criteria are met.

### Evaluating the resulting position

Before asking whether the resulting position is in a state of RE, we have to ask whether the process should even end at the point at which Thomson’s argumentation stops. Is it plausible that further instances of Steps A and B would not lead to an improved position?

***End Point Reached?*** On the system side, it might be possible to increase systematicity, e.g., by reducing the remaining three principles to another more general one. While it seems possible to further generalize P 2 and P 5 by eliminating the reference to concrete numbers, this would not change much. It would be more interesting to find a more general principle that would allow us to infer P 2, P 5, and P 6. However, there is no obvious candidate for such a principle—there might be such a candidate, but I don’t see any evidence for it based on the results of the reconstruction.

On the commitment side, making more commitments explicit would give more support to the current system, but as long as we assume that there are no further, conflicting commitments, this would not change much. Keeping in mind that any end point is always preliminary and can be revisited if new information arises, we can at least preliminarily follow Thomson and stop the process of adjustments here. But did we reach a position that is in a state of RE?

***RE reached?*** To assess whether a position in reflective equilibrium was reached, I use the RE conditions from Sect. 2 to analyze the resulting position.

*1. Are the resulting commitments and the system in agreement?* Yes—the resulting commitments and the resulting system are consistent and, given background information and background assumptions, the system supports the commitments. That is, each resulting commitment can be inferred from the system via an argument that includes additional background information and background assumptions.

*2. Do resulting commitments respect input commitments adequately?* In the initial position, we were only concerned with cases where we have to choose between the lives of five people against the life of one person. The resulting commitments also concern good deeds and altruistic behaviour. However, this does not mean that we changed the topic. Take into account that from the start, Thomson is also committed to the distinction between negative and positive duties (IC 5). All the new commitments concern cases where this distinction is seen as relevant. Thus, it is plausible that the subject was not abandoned. Adjustments to the input commitments—most importantly, the reversal of IC 3—could be justified given their relative weight in the position.

*3. Do at least some of the resulting commitments have independent credibility?* Assuming that the intuitions behind the commitments that Thomson adopts are not highly controversial, most of the resulting commitments have some positive degree of independent credibility. However, it is not clear that the intuitions and the reasoning behind Thomson’s commitments are uncontroversial. For example, Kamm (2016, p. 28) objects to the idea that we must not impose onto others what we are not willing to do ourselves: “We may sometimes draft someone to be a soldier even if we (and also he) would not and need not volunteer for service.” While commitments do not necessarily have to be completely uncontroversial, in order to fully defend her position Thomson would have to show how she can answer these objections. Or, if Thomson shares this commitment, it would turn out that her position is not, in fact, in agreement.

*4. Does the system do justice to theoretical virtues?* By introducing P 6, the *Negative Duties Vs. Positive Duties Principle* in Step $\hbox {A}_4$, the simplicity of the system could be increased as it needs fewer fundamental principles to support the commitments. By being able to support a range of different—and before seemingly disparate—commitments, P 6 also instantiates the virtue of having unificatory power. However, this also decreases the determinacy^10^ of the system. For example, it is less clear what it means that a negative duty “outweighs” a positive one, than what it means to rather let five die than kill one. Additionally, the reduction of the other principles to P 6 relies on a range of background assumptions which are not uncontroversial.

*5. Can the position be supported by background theories and background information?* Background theories were used neither to support nor attack the position. More work is necessary in order to show that the position is not only internally coherent, but also supported by background theories. Moreover, the resulting position relies heavily on some distinctions, e.g., between killing and letting die or what a “good deed” is (and what not). These distinctions are not uncontroversial, and it is not clear what exactly constitutes them. *6. Is the resulting position at least as plausible as available alternatives?* Alternatives were not developed, neither in Thomson (2008) nor as part of the reconstruction. Assessing the plausibility of the position as a whole would require us to go deeper into the trolley debate, to reconstruct alternative positions, and to compare them with respect to the RE criteria. This reconstruction merely represents a first step in this direction.

To sum up, the position meets some of the RE-criteria to a sufficient degree: the resulting system supports the resulting commitments, the resulting commitments respect the input commitments and have some independent credibility, and the system does justice to theoretical virtues. However, the analysis with the RE criteria also made salient a number of shortcomings: most importantly, that the position has not been explicitly related to background theories, its problematic reliance on background assumptions that are not further defended, that no real alternative candidates for parts of the system are assessed, and, closely connected with this, whether or not there are other positions that would meet the RE criteria to a higher degree.

Thus, I argue that at this point, no RE state was reached. The adjustment of IC 3 at Step $\hbox {B}_4$ can be defended with the RE-criteria, but that does not mean that the new NC 2 which replaces it is already justified as part of a position in RE.

## Discussion of results

The RE-conception described in Sect. 2 could be successfully applied as a method for reconstructing and analyzing Thomson’s arguments. Firstly, elements of RE such as commitments, parts of the system, and background information, could be clearly identified. It made sense to distinguish between input commitments (both initial and emerging) and commitments that were inferred from the system. And even though there was no explicit reference to something like theoretical virtues in Thomson’s argumentation, it was possible to reconstruct at least one step—$\hbox {A}_4$—where such considerations plausibly play a role.

Secondly, the RE-criteria provided real constraints for the process of adjustments: reversing the initial input commitment IC 3 was blocked in the beginning by the criterion that the independent credibility of commitments must not be discarded, but has to be outweighed by other considerations. In the initial position, no cogent argument could be made why the independent credibility of IC 3 should be outweighed. Also, reversing it would have amounted to rejecting a fifth of the current commitments—since we started with a narrow set of only five initial commitments. In the end, reversing the commitment could be vindicated because, based on a more fleshed-out position, a plausible argument could be given that the independent credibility of IC 3 is outweighed by the position as a whole.

Thirdly, not only was the RE conception applicable in the sense that its elements could be identified and its criteria applied but it was also possible to reconstruct Thomson’s arguments in a traceable step-by-step description as an RE-process, which allowed us to compare the input and resulting positions and to critically assess the latter. This does provide some support for the proposal to structure the RE-process along the lines of the two alternating kinds of steps A and B. This does not presuppose that Thomson was “actually” or “implicitly” doing RE all along, in the sense that she had this specific method in mind when making her arguments. For a successful reconstruction, we do not need to presuppose this.

Additionally, the reversal of IC 3 illustrates one of the particular benefits of the RE-conception that I adopted: our judgments on the various trolley cases are not included as intuitions, but as commitments. And our commitments can change while our intuitions might persist: it is entirely possible that I follow all of Thomson’s arguments, and am willing to commit to the new commitment NC 2—that the bystander must let the trolley continue in the two options case—while still having a strong *intuition* that he may let the trolley continue. In this RE framework, our intuitions are thus taken seriously, without having to be taken as fundamental: intuitions can provide independent credibility for commitments that enter the RE-process as input, but they are not themselves the input that has to be brought into equilibrium (for a more in-depth discussion of the role of intuitions for RE, see Brun, 2013).

While these results support the adopted RE-conception, the case study also provides insights for the further development and refinement of RE, as well as for its uses as a method. Most importantly, emerging commitments played a central role in the process. This shows how—at least for imperfect epistemic agents like us, who don’t have an overview of all of their relevant commitments—systematically exploring and broadening the set of commitments can be a part of the RE process and should be taken seriously by RE conceptions. Instead of describing RE exclusively as a process of adjustments between an already mostly explicit set of commitments and several candidate systems, we can also imagine it as starting from a specific problem which first needs to be spelled out and specified. Of course, it would be practical if the problem specification and description of the subject matter could be done before the RE-process starts. But this seems illusory, not only because of our limited ability to be aware of all our commitments. As the reconstruction shows, it is possible that changes to the system—like the introduction of P 5, the *No Major Altruism Required Principle*—would have implications for the current commitments that might lead us to explore further variations and making more emerging commitments explicit. And it is plausible that this can even be the case in situations where we put in a lot of effort to clarify the subject matter beforehand.

One might still object that the central role of the process and of emerging commitments is an artefact of the reconstruction, and that I could just as well have described the initial and the resulting position and compared them. However, as I have argued, even though adjusting the bystander commitment can be defended at Step $\hbox {B}_4$, no RE position was reached. One would need to continue the process and to flesh out the position in order to work towards an RE state. It is likely that the reasoning for adjusting IC 3 would be lost without documenting at least this aspect of the process. Describing the process is also part of making adjustments traceable and thereby enabling us to check whether the condition of respecting input commitments is met.

Also, it is important to keep in mind that in this case we have Thomson’s text, so we can trace how and why she added further elements. But this only holds for reconstructive uses, where we already know the “end point” of the process and need to reconstruct a way in which it could be reached via RE. Thus, this raises the question whether RE can actually be used as a “constructive” method to formulate and justify a position when starting from scratch. While this warrants its own case study, we can already draw some tentative conclusions.^11^ Constructive uses of RE are certainly more challenging than reconstructive ones—it is one thing to analyze a given position, and another thing entirely to make one’s own commitments explicit and to formulate principles from scratch. Nonetheless, it is plausible that RE can also be fruitfully applied in constructive situations. For example, the RE criteria can help us to structure our philosophical investigations. Analyzing a position with the RE criteria helps to identify problems and thereby sketches possible ways to move forward. In the case of “Turning the Trolley”, e.g., the analysis suggests that one of the next steps should be to focus on the distinctions of killing and letting die and the distinction between positive and negative duties in general. That this distinction is problematic might be clear even without the RE-reconstruction. But reconstructing the position and how it was reached with RE makes much more salient how the position as a whole could change depending on how one spells out the differences between the various cases.

Thus, by means of an example of restricted scope, I have demonstrated in this exploratory case study how one can use RE within the context of the trolley debate. I argue that in doing so, I have further demonstrated RE to be a powerful methodological framework that is worth adopting. Of course, in order to achieve a comprehensive position on trolley cases in RE, focusing on just one paper by one author is far from enough. Whoever wanted to undertake such a task would have to consider and include further proposals and commitments that are part of the subject matter. This could mean both consulting further written works as well as using empirical studies to explore judgments of, e.g., lay people (see Awad et al., 2018 for an example of such a study).

Let me conclude this discussion by stressing another point about RE: as the reconstruction shows, a lot depends on the cases that are investigated, as well as on the principles that we can come up with. Thus, for us as imperfect epistemic agents, a lot depends on whether we come up with the “right” scenarios, examples, and principles. The more we allow others to challenge our positions, the more likely it is that we can avoid such problems. This suggests that to avoid accidental generalizations, RE processes conducted by agents with a bounded rationality need a social dimension.^12^

## Conclusion

In this paper, I conducted a case study on a specific conception of reflective equilibrium (RE) by reconstructing a paper from the trolley debate as an RE-process. In doing so, I was able to demonstrate:

Firstly, there is at least one conception of RE (as described in Sect. 2) that is sufficiently specified to be applicable.

Secondly, the RE conception was not only applicable in the sense that Thomson’s arguments could be reconstructed with it, but its criteria also turned out to provide real constraints on the process of justification. For example, the criterion that input commitments need to be respected first blocked the adjustment of the bystander commitment, but ultimately—after some adjustments—vindicated it.

Thirdly, an explicit application of RE has benefits in terms of clarity, e.g., for identifying weak parts of the position. At the same time, the RE criteria provide guidance for how the process could continue and the position be improved. With respect to “Turning the Trolley”, this could mean taking a closer look at the background, and scrutinizing the presupposed distinctions.

And lastly I have argued that RE is not only a helpful tool for the reconstruction of individual parts of the trolley debate, but that it provides a powerful methodological framework for the discussion (not only) of trolley cases more general.

## Appendix A: Elements of the RE process

This appendix lists all the elements of the reconstructed RE-process. Together with Fig. 3 (p. 17), it can be used to supplement the description in the main text to trace the whole process in detail from initial to resulting position. All principles and their specifications are listed in A.1, commitments—initial, emerging, and newly inferred ones—in 1, and case descriptions in A.3. Additions that were made as part of the reconstruction are marked as such.

### A.1: Principles

- $P\,1:$  *Letting Five Die Vs. Killing One Principle* A must let five die if saving them requires killing B (Thomson, 2008, p. 360). [Part of the Initial Position]
- $P\,1.1:$  *Letting Five Die Vs. Killing One Principle, Non-Altruist’s Version* A must not kill B to save five if A were not willing to kill A-self to save five given the option (see Thomson, 2008, p. 366; not a principle in the original text). [Variation of P 1, added at Step $\hbox {A}_2$]
- $P\,1.2:$ *Letting Five Die Vs. Killing One Principle, Altruist’s Version* A must no kill B to save five even if A were willing to kill A-self to save five given the option (see Thomson, 2008, p. 367; not a principle in the original text.) [Variation of P 1, added at Step $\hbox {A}_3$]
- $P\,2:$ *Killing Five Vs. Killing One Principle* A must not kill five if A can instead kill one (Thomson, 2008, p. 360). [Part of the Initial Position]
- $P\,3:$ *Paying For Your Own Good Deeds Principle* A must not make B pay the cost of a good deed that A wants to do if A can instead pay the cost A-self. (Thomson, 2008, p. 365, N.B.: in the original text, this is not introduced as a *principle*, but rather as a general remark that supports introducing the principle that in the reconstructed process is P 4.) [Added at Step $\hbox {A}_1$]
- $P\,3.1:$ *First Modified Paying For Your Own Good Deeds Principle* A must not make B pay the cost of a good deed that (i) A wants to do, but (ii) A cannot pay the cost A-self, if A would not be willing to pay the cost A-self [re-constructional addition made by T.R., not in Thomson (2008)]. [Variation of P 3, added at Step $\hbox {A}_2$]
- $P\,3.2:$ *Second Modified Paying For Your Own Good Deeds Principle* A must not make B pay the cost of a good deed that (i) A wants to do, (ii) A cannot pay the cost A-self, (iii) A would be willing to pay the cost A-Self if possible, if B is not required to pay the cost [re-constructional addition made by T.R., not in Thomson (2008)]. [Variation of P 3, added at Step $\hbox {A}_3$]
- $P\,4:$ *Letting Five Die Vs. Killing One Vs. Killing Yourself Principle* A must not kill B to save five if A can instead kill A-self to save the five (Thomson, 2008, p. 365, N.B.: In the original text it is called the *Third Principle*). [added at Step $\hbox {A}_1$]
- $P\,5:$ *No Major Altruism Required Principle* One is not required to give up one’s life upon learning that five others will live if and only if oneself dies (Thomson 2008, p. 365, N.B.: In the original text it is called the *Fourth Principle*). [added at Step $\hbox {A}_1$]
- $P\,6:$ *Negative Duties Vs. Positive Duties Principle* When a negative and a positive duty conflict, fulfilling the negative duty outweighs the positive duty (see (Thomson, 2008), pp. 372–374, not explicitly introduced as a principle in the original text). [added at Step $\hbox {A}_4$]

### A.2: Commitments

#### A.2.1: Initial input commitments

- *IC* 1 In Case 1, the judge must not frame an innocent person and have them executed to save five hostages (Thomson, 2008, p. 359).
- *IC* 2 In Case 2, the driver of a runaway trolley must divert his trolley so that one workman dies instead of five (Thomson, 2008, p. 360).
- *IC* 3 In Case 3, the bystander at the switch may divert the trolley so that one workman dies instead of five (Thomson, 2008, p. 361). **[conflicts with P 1]**[Replaced by NC 2 at Step $\hbox {B}_4$]
- *IC* 4 In Case 4, the bystander on the bridge must not shove the fat man in front of the trolley in order to save the five workmen on the track (Thomson, 2008, p. 362).
- *IC* 5 There is a distinction between what we owe to people in the form of aid (positive duties) and what we owe to people in the way of non-interference (negative duties). Negative duties are markedly weightier than positive duties (Thomson, 2008, p. 360).

#### A.2.2: Emerging input commitments

- *EC*1 The bystander in Case 5, *Bystander’s Three Options*, must not divert the trolley onto the single workman (Thomson, 2008, p. 364). [Emerges at Step $\hbox {B}_1$]
- *EC*2 I must not steal money to make a donation to Oxfam in Case 6, *Oxfam Donation* (Thomson, 2008, p. 365). [Emerges at Step $\hbox {B}_1$]
- *EC*3 If A wants to do a certain good deed, and discovers that the only permissible means he has of doing the good deed is killing himself, then he may refrain from doing the good deed (Thomson, 2008, p. 365). [Emerges at Step $\hbox {B}_1$]
- *EC*4 In Case 5, *Bystander’s Three Options*, the bystander may let the trolley continue onto the five workmen (Thomson, 2008, p. 365). [Emerges at Step $\hbox {B}_1$, supported by EC 3]
- *EC*5 You must not steal money to make a donation to Oxfam in Case 6.1, *First Modified Oxfam Donation*. [Emerges at Step $\hbox {B}_2$, addition made by T.R., not in Thomson (2008).]
- *EC*6 A non-altruistic bystander must not divert the trolley onto the single workman in Case 3, *Bystander’s Two Options* (Thomson, 2008, p. 367). [Emerges at Step $\hbox {B}_2$]
- *EC*7 You must not steal money to make a donation to Oxfam in the Case 6.2, *Second Modified Oxfam Donation*. [Emerges at Step $\hbox {B}_3$, addition made by T.R., not in Thomson (2008).]
- *EC*8 An altruistic bystander must not divert the trolley onto the single workman in Case 3, *Bystander’s Two Options* (Thomson, 2008, pp. 366–367). [Emerges at Step $\hbox {B}_2$]

#### A.2.3: Newly inferred commitments

- *NC*1 The single workman on the track in *Bystander’s Two Options* is not required to consent to having the trolley turned onto him so that the five can be saved (Thomson, 2008, p. 367). [Inferred with P 5]
- *NC*2 The bystander in *Bystander’s Two Options* must not divert the trolley onto the single workman (Thomson, 2008, pp. 367–368). [Replaces IC 3] [Inferred with P 1]

### A.3: Background

#### A.3.1: Case descriptions

- *Case 1: Judge’s Two Options* A crime has been committed, and some rioters have taken five innocent people hostage; they will kill the five unless the judge arranges for the trial, followed by the execution, of the culprit. The real culprit is unknown. The judge has only two options: He can (i) let the rioters kill the five hostages, or (ii) frame an innocent person for the crime, and have him executed (Thomson, 2008, p. 359).
- *Case 2: Trolley Driver’s Two Options* The driver of a runaway trolley has only the following two options: He can (i) continue onto the track ahead, on which five men are working, and five die, or (ii) steer onto a spur of track off to the right on which only one man is working, and one dies (Thomson, 2008, p. 360).
- *Case 3: Bystander’s Two Options* The driver of a runaway trolley has dropped dead of a heart attack. A bystander happens to be standing by the track, next to a switch that can be used to turn the trolley off the straight track, on which five men are working, onto a spur of track to the right on which only one man is working. The bystander has only two options: He can (i) do nothing, and five die, or (ii) throw the switch, and one dies (Thomson, 2008, p. 361).
- *Case 3.1: Non-Altruistic Bystander’s Two Options* The driver of a runaway trolley has dropped dead of a heart attack. A bystander happens to be standing by the track, next to a switch that can be used to turn the trolley off the straight track, on which five men are working, onto a spur of track to the right on which only one man is working. The bystander has only two options: He can (i) do nothing, and five die, or (ii) throw the switch, and one dies. If the bystander had the option of turning the trolley onto himself to save the five, he would not do it (Thomson, 2008, p. 365).
- *Case 3.2: Altruistic Bystander’s Two Options* The driver of a runaway trolley has dropped dead of a heart attack. A bystander happens to be standing by the track, next to a switch that can be used to turn the trolley off the straight track, on which five men are working, onto a spur of track to the right on which only one man is working. The bystander has only two options: He can (i) do nothing, and five die, or (ii) throw the switch, and one dies. If the bystander had the option of turning the trolley onto himself to save the five, he would do it (Thomson, 2008, p. 366).
- *Case 4: Fat Man* A bystander is standing on a footbridge over a trolley track, and sees a trolley hurtling down the track, out of control. He sees that five workmen are on the track where it exits from under the footbridge. Next to him stands a very fat man. The bystander has only two options: He can (i) do nothing, the trolley continues on his track, and five die, or (ii) shove the fat man over the bridge, stopping the trolley, and one would die (Thomson, 2008, p. 362).
- *Case 5: Bystander’s Three Options* The driver of a runaway trolley has dropped dead of a heart attack. A bystander happens to be standing by the track, next to a switch that can be used to turn the trolley off the straight track, on which five men are working. The bystander has only three options: He can (i) do nothing, and five die, (ii) throw the switch to the right, and the one workman on the right hand track dies, or (iii) throw the switch to the left, on the spur of track he is standing on himself, and he will die (Thomson, 2008, p. 364).
- *Case 6: Oxfam Donation* I am asked for a donation to Oxfam. I want to send some money, and would be able to do so. But I don’t feel like sending them my own money. Is it permissible for me to steal from someone else and send that money to Oxfam (Thomson, 2008, p. 365)?
- *Case 6.1: First Modified Oxfam Donation* I am asked for a donation to Oxfam. I want to send some money, but don’t have any of my own. However, even if I had the money, I would not be willing to give it. I just want Oxfam to receive the donation. Is it permissible for me to steal from someone else and send that money to Oxfam? [Variation of Case 6, added by T.R. as part of the reconstruction.]
- *Case 6.2: Second Modified Oxfam Donation* I am asked for a donation to Oxfam. I want to send some money, but don’t have any of my own. If I had the money myself, I would send it. Is it permissible for me to steal from someone else and send that money to Oxfam? [Variation of Case 6, added by T.R. as part of the reconstruction.]

## Footnotes

1 I don’t want to deny, for example, that Rawls (1999) uses reflective equilibrium. But his focus is on developing a theory of justice, and the application of RE and its criteria is not made explicit and traceable.

2 This paper focuses on how RE can be used to rationally reconstruct and evaluate a philosophical position. For a case study on how RE can be used as a constructive method, i.e., for the development of a new position, see Rechnitzer (2022).

3 For what I call “input commitments”, typically “antecedent” or “initial” is used in the literature. I am avoiding “antecedent” since it suggests that commitments were just held at some prior point, but not necessarily before or independently of the RE-process. And I use “initial” as a subgroup of input commitments, to distinguish them from other input commitments that only get explicitly considered when the process is already under way. See also Baumberger and Brun (2021, p. 7932).

4 Whether Thomson is correct in this assessment is an open question at this point. But currently, we are still reconstructing her arguments. They will be critically evaluated in Sect. 4.

5 These options are discussed in Thomson (1985).

6 Because of this reference to good deeds, I introduced a modification of *Oxfam Donation* in the reconstruction (see appendix: Case 6.1, *First Modified Oxfam Donation*, EC 5). While Thomson does not explicitly consider this, it is helpful for the clarity of her argument. In *First Modified Oxfam Donation*, you would like to donate money to Oxfam, but don’t have enough of your own. However, even if you had the money, you would not be willing to give it. You just want Oxfam to have the money. Is it permissible for you to steal money from someone else to donate it? I expect that Thomson would be committed to a negative answer, and that she would see this case as analogous to *Non-Altruistic Bystander’s Two Options*.

7 And of course also the workman in *Bystander’s Three Options*.

8 That Thomson now states that letting the trolley continue is a permissible option for the bystander at the switch reveals that IC 3 must be read in the sense that it is permissible, but not obligatory, for the bystander to turn the trolley.

9 Alternatively, P 1.2 can directly support EC 8, but if we accept that saving five is a good deed etc., then P 1.2 is something like a “shorthand” for the application of P 3.2, and making the argument with P 3.2 makes the reasoning more explicit.

10 In the sense of Timmons (2012, p. 13): “A moral theory should feature principles which, together with relevant factual information, yield determinate moral verdicts about the morality of actions, persons, and other objects of evaluation in a wide range of cases.”

11 For a case study on such a constructive use of RE, see Rechnitzer (2022).

12 For a discussion of social dimensions of RE, see Elgin (1996, pp. 111–119) and Baderin (2017).
